# Noisy Galvanic Vestibular Stimulation (Stochastic Resonance) Changes Electroencephalography Activities and Postural Control in Patients with Bilateral Vestibular Hypofunction

**DOI:** 10.3390/brainsci10100740

**Published:** 2020-10-15

**Authors:** Li-Wei Ko, Rupesh Kumar Chikara, Po-Yin Chen, Ying-Chun Jheng, Chien-Chih Wang, Yi-Chiang Yang, Lieber Po-Hung Li, Kwong-Kum Liao, Li-Wei Chou, Chung-Lan Kao

**Affiliations:** 1 Institute of Bioinformatics and Systems Biology, National Chiao Tung University, Hsinchu 300, Taiwan; lwko@nctu.edu.tw (L.-W.K.); rupesh.bt01g@g2.nctu.edu.tw (R.K.C.); 2 Department of Biological Science and Technology, National Chiao Tung University, Hsinchu 300, Taiwan; 3 Center for Intelligent Drug Systems and Smart Bio-Devices (IDS2B), National Chiao Tung University, Hsinchu 300, Taiwan; 4 Drug Development and Value Creation Research Center, Kaohsiung Medical University, Kaohsiung 807, Taiwan; 5 Department of Physical Therapy and Assistive Technology, National Yang-Ming University, Taipei 112, Taiwan; azxd32@gmail.com (P.-Y.C.); cycom1220@gmail.com (Y.-C.J.); 6 Department of Physical Medicine and Rehabilitation, Taipei Veterans General Hospital, Taipei 112, Taiwan; yichiang2312@hotmail.com; 7 School of Medicine, National Yang-Ming University, Taipei 112, Taiwan; phli@gm.ym.edu.tw; 8 Department of Physical Medicine and Rehabilitation, Taipei Veterans General Hospital Yuli Branch, Hualien 98142, Taiwan; candycandywang@gmail.com; 9 Institute of Clinical Medicine, National Yang-Ming University, Taipei 112, Taiwan; 10 Department of Otolaryngology, Cheng Hsin General Hospital, Taipei 112, Taiwan; 11 Department of Neurology, Neurological Institute, Taipei Veterans General Hospital, Taipei 112, Taiwan; kkliao0730@gmail.com

**Keywords:** electroencephalography (EEG), independent component analysis (ICA), galvanic vestibular stimulation (GVS), bilateral vestibular hypofunction (BVH)

## Abstract

Patients with bilateral vestibular hypofunction (BVH) often suffer from imbalance, gait problems, and oscillopsia. Noisy galvanic vestibular stimulation (GVS), a technique that non-invasively stimulates the vestibular afferents, has been shown to enhance postural and walking stability. However, no study has investigated how it affects stability and neural activities while standing and walking with a 2 Hz head yaw turning. Herein, we investigated this issue by comparing differences in neural activities during standing and walking with a 2 Hz head turning, before and after noisy GVS. We applied zero-mean gaussian white noise signal stimulations in the mastoid processes of 10 healthy individuals and seven patients with BVH, and simultaneously recorded electroencephalography (EEG) signals with 32 channels. We analyzed the root mean square (RMS) of the center of pressure (COP) sway during 30 s of standing, utilizing AMTI force plates (Advanced Mechanical Technology Inc., Watertown, MA, USA). Head rotation quality when walking with a 2 Hz head yaw, with and without GVS, was analyzed using a VICON system (Vicon Motion Systems Ltd., Oxford, UK) to evaluate GVS effects on static and dynamic postural control. The RMS of COP sway was significantly reduced during GVS while standing, for both patients and healthy subjects. During walking, 2 Hz head yaw movements was significantly improved by noisy GVS in both groups. Accordingly, the EEG power of theta, alpha, beta, and gamma bands significantly increased in the left parietal lobe after noisy GVS during walking and standing in both groups. GVS post-stimulation effect changed EEG activities in the left and right precentral gyrus, and the right parietal lobe. After stimulation, EEG activity changes were greater in healthy subjects than in patients. Our findings reveal noisy GVS as a non-invasive therapeutic alternative to improve postural stability in patients with BVH. This novel approach provides insight to clinicians and researchers on brain activities during noisy GVS in standing and walking conditions in both healthy and BVH patients.

## 1. Introduction

Vestibular systems sense linear and angular movements of the head, keeping the body in an upright position to maintain gaze and postural control. Through the peripheral vestibular organs, i.e., the semicircular canals and otoliths, vestibular afferents continuously provide precise information to the brain, so that individuals can explore the environment without losing balance [1]. Patients with bilateral vestibular hypofunction (BVH) often experience a variety of symptoms, including dizziness, oscillopsia, spatial disorientation, and unsteadiness during standing and walking [2,3]. Until now, the primary treatment for BVH has been physical therapy, with the optimization of its efficacy becoming an eminent issue, as patients with BVH suffer from a higher risk of falls.

The vestibular system is known to exhibit high neuroplasticity [4]. Although there is no primary cortical area responsible for vestibular functions, the parieto-insular-vestibular-cortex is known to be the most robust area modulating the vestibular system [5,6]. By applying galvanic vestibular stimulation (GVS), the firing activity of the eighth cranial nerve is enhanced on the side with the cathode electrode and decreased on the side with the anode electrode [7,8]. This current input is a non-invasive method that has long been applied in the investigation of vestibular functions. Along with functional imaging tools, such as functional Magnetic Resonance Imaging (fMRI) and electroencephalography (EEG), GVS over both mastoids has helped scientists to reveal the complex network of the vestibular system. Only recently, zero-mean GVS current delivered to the mastoids has been used to improve the postural control of patients with BVH and has gained growing attention. Zero-mean GVS, also called noisy GVS (or stochastic resonance, SR) has been shown to improve the function of detection in sensory neurons by reduce the threshold of sensory input [9,10,11]. Noisy GVS has been applied in various patient populations to improve neuroplasticity, e.g., in enhancement of spatial memory, development of motor control in patients with Parkinson disease, improvement of cognitive deficiencies in those with Alzheimer’s disease, and improvement of recovery of visual deficits in patients after stroke. In addition, it has been previously applied in patients with vestibular disorders and in healthy subjects [1,12,13], and shown to reduce center of pressure (COP) sway in patients with bilateral vestibulopathy [13]. Unlike caloric testing or conventional GVS, noisy GVS provides stimulation without directional specificity, acting as a perfect means for enhancing vestibular sensory inputs in both vestibular afferents in patients with BVH. There is a growing body of literature exploring the clinical applications of noisy GVS for improving static and dynamic postural control in these patients [1,14]. The applications of this novel methodology have been expanded by the discovery of a non-invasive prosthetic device used for rehabilitation of patients with BVH.

EEG signals clean from artifacts may be obtained by independent component analysis (ICA) methods, which separate the various types of artifact. An earlier study using ICA processing investigated the amplitude modulations of EEG signals associated with gait. During active walking, the upper alpha (10–12 Hz) and beta (18–30 Hz) oscillations in the central sensorimotor areas of the brain were suppressed compared to those during the standing condition [15]. Another ICA study reported that EEG beta band activity in the premotor cortex is higher during stabilized than during normal gait [16]. To date, the cortical effects of noisy GVS during walking have not been thoroughly investigated, while changes in neural activities after noisy GVS are still unclear. Therefore, the aim of this study was to observe and compare the differences in cortical stimulation mappings during standing and walking with head turning, before and after the noisy GVS, in order to elucidate the underlying neural mechanisms. To this end, we utilized an EEG neuroimaging method that detects neuromodulations in the human brain in the above-mentioned conditions.

## 2. Materials and Methods

### 2.1. Participants

In this prospective, observational study, we recruited healthy subjects as well as BVH patients from hospital. The BVH patients were recruited from the Department of Physical Medicine and Rehabilitation, Taipei Veterans General Hospital as well as Department of Otolaryngology, Cheng Hsin General Hospital. The patients presented to ENT clinic with complaints of dizziness/vertigo, oscillopsia or unsteady gait. The ENT doctor (Liber PH Li) performed the Caloric test to confirm the diagnosis of bilateral vestibular hypofunction. The patients were then referred to PM&R (CL Kao) to receive the video head impulse test (vHIT) to evaluate the vestibular ocular reflex (VOR) gains. The diagnosis of BVH was based on the results of air irrigation caloric test as well as vHIT results. A total response in the caloric test <20 degrees per second was defined as BVH. The gain of VOR in healthy people ranges from 0.9 to 1.0. VOR gain less than 0.9 on either side is considered hypofunction

The exclusion criteria included any history of neurological or orthopedic disorders, and any visual, auditory or cognitive impairments. At the beginning of the trial, each participant signed an informed consent, approved by the Ethics Committee of Taipei Veterans General Hospital and the Food and Drug Administration, Taiwan, Republic of China. (trial no. NCT0355494).

### 2.2. Experimental Design

**Figure 1:**
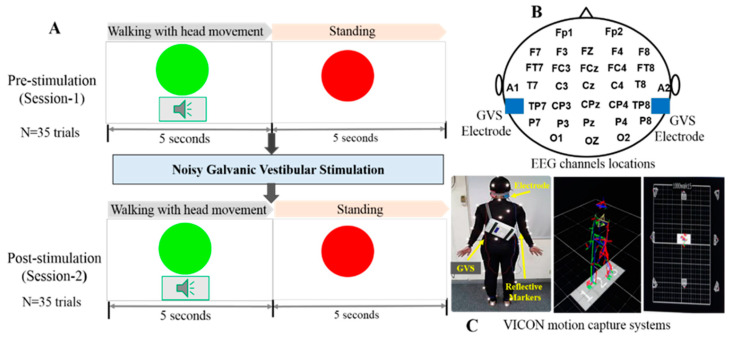
Experimental design. (**A**) Presentation of green and red circle stimuli for walking and standing in pre-stimulation and post-stimulation conditions. (**B**) Placement of electroencephalography (EEG) electrodes. EEG cap with 32 channels was placed on the scalp according to the International 10–20 System. Galvanic vestibular stimulation (GVS) electrodes were placed with one electrode on the mastoid process behind each ear denoted by blue square. (**C**) VICON motion capture (Vicon Motion Systems Ltd., Oxford, UK) process: Subjects wore standard motion capture suits with plug-in-gait model setting. (Left and middle figures). The wireless transcranial direct-current stimulation (tDCS) box was fixed on the subjects’ backs. The subjects were tested in the capture volume with eight cameras (right figure).

We designed an experimental scenario to examine neural activity changes in healthy controls and patients with BVH under both a walking and a standing condition, and in relation to noisy GVS (Figure 1A). In each trial, a green “circle” symbol and an alarm sound (65 dB, 500 Hz) were presented to instruct subjects to perform 5 s of walking with horizontal head movement. As they walked, they were instructed to turn their head horizontally every 500 ms at the speed of 2 Hz according to the auditory cues, since the vestibular system was previously shown to play a role in 2 Hz head yaw movements [17]. Then, a red “circle” symbol was presented to instruct the subjects to perform 5 s of standing. We projected all stimuli onto a screen in front of the participants for them to perceive the instructions more easily during the trial conditions. Each participant was required to complete 70 trials (35 trials pre-stimulation, and 35 trials post-stimulation). The entire experimental process for each participant lasted approximately one hour. The placement of EEG electrodes is shown in Figure 1B.

A VICON computer-assisted video motion analysis system (VICON Motion Systems, Oxford, UK) was utilized for motion capture in this experiment. For studying the motion trials, we used VICON standard mode (Full body modeling with Plug-in Gait), with 8 MX T-020 VICON cameras (Vicon Motion Systems Ltd., Oxford, UK) and one AMTI force plate (Advanced Mechanical Technology Inc., Watertown, MA, USA) in a 6 × 10 m space. Subjects wore standard motion-capture suits with a transcranial direct-current stimulation (tDCS) main box fixed on their back, as appropriate for capturing the light markers (Figure 1C). All signals were simultaneously recorded by a personal computer (PC) with a signal sampling rate of 100 Hz. To determine the optimal GVS intensity for each subject, a range of stimulation strengths was used (peak amplitude 0, 200, 400, 600, 800, or 1000 µA). When subjects received noisy GVS with different random intensities, they were asked to stand on the AMTI force plate as steadily as possible for 30 s. After recording the COP (X, Y) in the standing position, we averaged all COP positions during that period of standing and calculated the root mean square (RMS) from the averaged (X, Y) point. Then, we compared the COP RMS under different stimulations to determine the minimal value and defined that value as the optimal intensity, since higher RMS values indicate worse standing stabilities. Each subject then walked with a 2 Hz head yaw, with and without noisy GVS. The 2 Hz frequency was provided by a metronome, and the subjects were requested to make their best effort to keep up with the rhythm. The VICON system captured each subject’s corresponding gait motion.

### 2.3. Noisy Galvanic Vestibular Stimulation Process

Noisy GVS was provided using DC-STIMULATOR PLUS (Eldith, NeuroConn GmbH, Ilmenau, Germany). During the stimulation period, noisy GVS was delivered at its optimal intensity for 6 min in the walking and standing conditions, through carbon rubber electrodes, bilaterally and bipolarly. An electrode coated with Tac gel was placed over the mastoid process, behind each ear, to optimize conductivity and adhesiveness. Analog command voltage signals were subsequently passed to a constant current stimulator connected to the stimulation electrodes, as described previously [12,13,18].

### 2.4. EEG Acquisition and Analysis

The EEG signals were acquired from all subjects using a 32-channel EEG cap connected to a neuro Scan NuAmps system (Compumedics USA Inc., Charlotte, NC, USA). They were down-sampled from 1000 to 250 Hz and filtered through a 1 to 50 Hz band-pass finite impulse response (FIR) filter using the EEGLAB toolbox (Version 13.6.5b, UC San Diego, Swartz Center for Computational Neuroscience (SCCN), La Jolla, CA, USA. [16,19]. Then, they were re-referenced to the mean of the A1 and A2 electrodes before further event-related spectral perturbation (ERSP) analysis [20]. EEG signals were analyzed by MATLAB R2014 (The MathWorks Inc., Natick, MA, USA).

In this study, ICA was used to separate brain region dipole sources of brain activity during walking and standing [21]. The extracted EEG signals were analyzed using time-frequency analysis of ERSP [21,22]. Seven clusters with independent components from all subjects were selected for ERSP analysis. After ICA processing, each dipole source was investigated using DIPFIT2 routines in EEGLAB, to find the 3D location of an equivalent dipole source, based on a four-shell spherical head model [16,19]. After manually removing the artifact components, component clustering was performed using k-means (k = 7) criteria and dipole-fitting coordinates to identify the most representative clusters [19]. The value of k was obtained to select the independent components that were found before and after GVS.

After completing EEG-ICA pre-processing, each epoch was extracted from −2 to 8 s (i.e., baseline −2 to 0 sec; walking 0 to 5 sec.; standing 5 to 8 sec) before and after noisy GVS. From the resting condition (i.e., standing position), we used 2 s EEG signals as baseline in each trial. The power spectrum of EEG signals was divided into five frequency bands, delta (1–4 Hz), theta (4–8 Hz), alpha (8–12 Hz), beta (13–30 Hz), and gamma (30–50 Hz), in order to observe the changes in neural activity before and after the noisy GVS.

### 2.5. Statistical Analysis

In the VICON experiment, the COP in the XY-plane and the RMS of the trajectory to the mean point were measured [17]. During the 2 Hz head yaw walking task, we measured the absolute difference of the subjects’ head rotation. The differences in COP RMS and head rotation velocity with and without GVS were compared using Wilcoxon signed-rank test, with alpha set at 0.05. Statistically significant (*p* < 0.05) differences before and after noisy GVS in ERSP analysis were estimated using bootstrap statistical processing [23] in the EEGLAB toolbox [19]. To calculate multiple comparisons, the significance values were corrected using the false discovery rate (FDR) method [24] in EEGLAB [19].

## 3. Results

### 3.1. Demographic Data

Ten healthy participants (seven male, three female; age range 23–53 years, mean ± SD age: 29.1 ± 8.4 years) and seven patients with BVH (all female; age range 22–68 years, mean ± SD age: 53.4 ± 15.7 years) completed the trials. The subjects’ demographic data is shown in Table 1.

### 3.2. Behavioral Results

**Figure 2:**
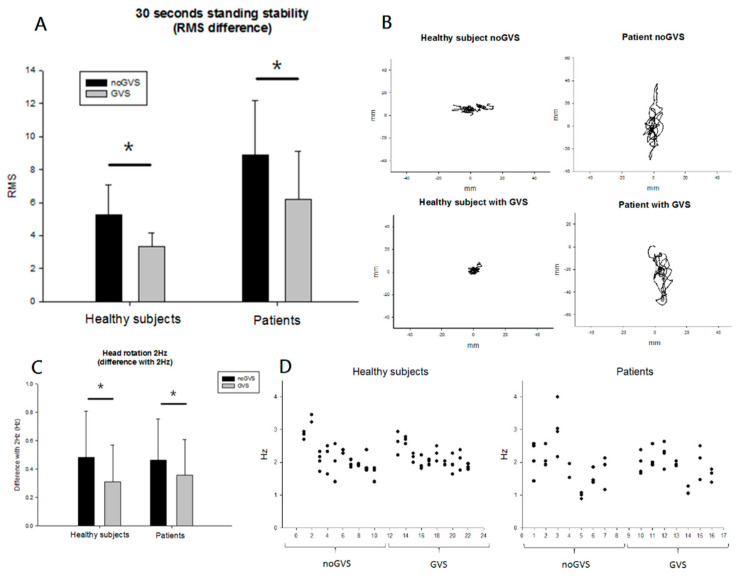
Behavioral results of subjects with and without noisy galvanic vestibular stimulation (GVS) during walking and standing conditions. (**A**) Shows the center of pressure (COP) displacements mean root mean square (RMS) in healthy and patient groups. In healthy group, the mean RMS was 5.27 ± 1.79 without noisy GVS and 3.36 ± 0.80 with noisy GVS in standing condition (*p* = 0.005, Wilcoxon signed rank test). In patient group, the mean RMS was 8.86 ± 3.31 without noisy GVS and 6.19 ± 2.29 with noisy GVS (*p* = 0.018, Wilcoxon signed rank test). (**B**) Displays the COP displacements in one single healthy & one patient subjects. When walking with 2 Hz head yaw, both healthy and patient groups showed a tendency towards a closer proximity to 2 Hz head yaw with noisy GVS. (Healthy subjects with GVS = 0.48 ± 0.32 Hz; without GVS = 0.31, ± 0.26 Hz, *p* = 0.005. In patients, with GVS = 0.46, ± 0.28 Hz; without GVS = 0.35, ± 0.24 Hz, *p* = 0.018, (**C**). The head turning frequency for each subject with and without noisy GVS is shown in scattered plot (**D**). * no GVS: the condition without noisy GVS stimulation. * GVS: the condition with noisy GVS.

In the healthy group, the mean RMS displacement was 5.27 ± 1.79 mm RMS of averaged COP point without noisy GVS and 3.36 ± 0.80 mm RMS of mean COP with noisy GVS for the standing condition (*p* = 0.005, Wilcoxon signed rank test). In the patient group, the mean RMS was 8.86 ± 3.31 mm and 6.19 ± 2.29 mm without and with noisy GVS, respectively (*p* = 0.018, Wilcoxon signed rank test, Figure 2A). A plot can be seen of typical COP displacements over the course of a trial for two individual subjects (one healthy subject and one patient, Figure 2B).

When walking with a 2 Hz head yaw, both groups showed a closer approximation to the 2 Hz head yaw with noisy GVS than without (healthy subjects with GVS: 0.48 ± 0.32 Hz, without GVS: 0.31 ± 0.26 Hz, *p* = 0.005; patients with GVS: 0.46 ± 0.28 Hz, without GVS: 0.35 ± 0.24 Hz, *p* = 0.018; Figure 2C). The head turning frequency for each subject, with and without noisy GVS, is shown in the form of scatter plots in Figure 2D. These behavioral results indicated that the RMS of COP sway was significantly reduced during noisy GVS in patients and healthy subjects while standing. In addition, both groups showed significantly better control in performing the 2 Hz head yaw movements with noisy GVS while walking.

### 3.3. EEG Results: EEG Scalp Map and Dipole Source Locations

**Figure 3:**
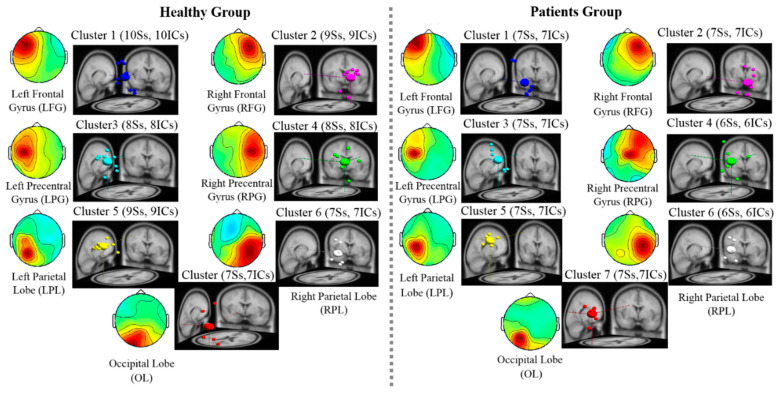
Independent component clusters of dipole locations for the analysis of brain dynamics. The activated seven clusters in healthy subjects and patients with bilateral vestibular hypofunction (BVH) were identified including left frontal gyrus (LFG), right frontal gyrus (RFG), left precentral gyrus (LPG), right precentral gyrus (RPG), left parietal lobe (LPL), right parietal lobe (RPL) and occipital lobe (OL).

The independent components (IC) obtained from all subjects with similar scalp maps and dipole source locations, clustered into the same groups, is shown in Figure 3. The value of k was obtained by considering the potential number of dipole sources activated after GVS in the walking and standing conditions. The seven clusters of IC in Figure 3 were common among all subjects [25,26]. The seven activated brain regions were identified as left and right frontal gyrus, left and right precentral gyrus (LPG and RPG, respectively), left and right parietal lobe (LPL and RPL, respectively), and occipital lobe (OL). The activation of these seven brain regions verifies that the resulting mean of the independent components within each cluster was highly similar for each scalp map and dipole source location (Figure 3).

### 3.4. Noisy GVS Increases EEG Activities in Patients with BVH and Healthy Subjects

**Figure 4:**
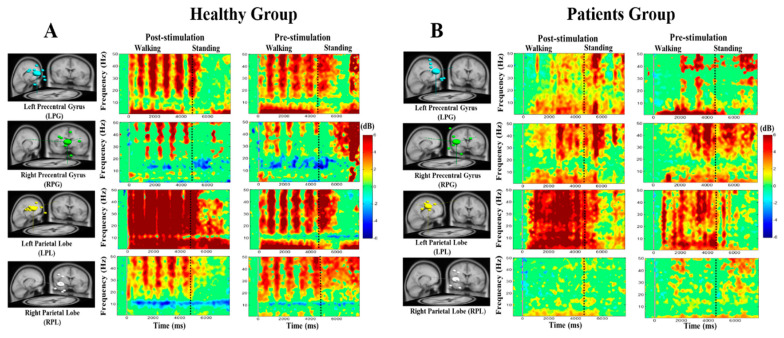
(**A**) The event-related spectral perturbation (ERSP) images of healthy subjects in left and right precentral gyrus (LPG, RPG), and left and right parietal lobe (LPL and RPL) of the brain. (**B**) The ERSP of patients with BVH in LPG, RPG, LPL and RPL of the brain after GVS stimulation. The vertical x-axis reveals the frequency (Hz), horizontal y-axis shows the time (ms), color bars indicate the power (dB) of the ERSPs, in which red indicates significant power increase and green shows power decrease relative to baseline (false discovery rate (FDR)-adjusted, *p* < 0.05); statistical threshold at *p* < 0.05.

In the EEG study, we investigated the average ERSP during the standing condition in patients with BVH and healthy subjects after noisy GVS. EEG activities were observed in the LPG, RPG, LPL, and RPL during pre- and post-stimulation conditions in healthy subjects and patients. For the standing condition in healthy subjects and patients, beta and gamma band activities at the LPG, RPG, LPL, and RPL increased more after than before the stimulation (Figure 4A,B).

The mu rhythm and alpha power in the LPG, RPG, LPL, and RPL were suppressed during post-stimulation and pre-stimulation during walking both in healthy subjects and patients (Figure 4A,B). This suppression is related to movement of the feet or legs. However, we observed less mu rhythm suppression in patients than in healthy controls.

During head turning in healthy controls and patients, the power of theta, beta, and gamma bands increased more after than before the stimulation at the LPG, RPG, LPL, and RPL (Figure 4A,B). The EEG activities increased during head turning possibly because of the neuroplasticity induced by noisy GVS in these vestibular regions.

**Figure 5:**
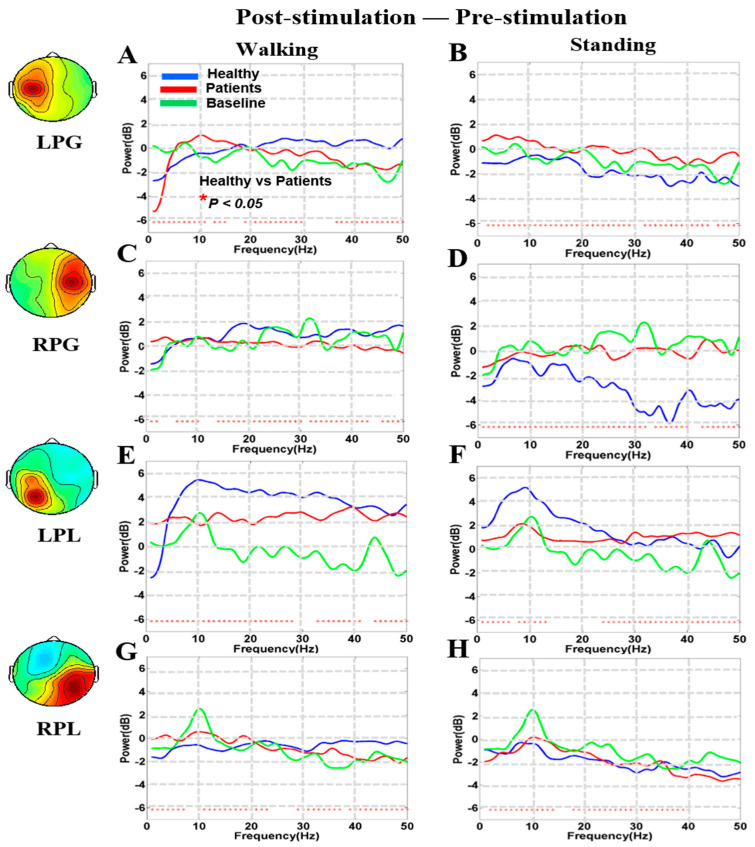
The power spectral density results of the brain with healthy and BVH patients. These result in LPG, RPG, LPL, and RPL shows the effect of noisy GVS (post-stimulation–pre-stimulation) in healthy vs. patients during walking and standing conditions. The power spectra of patients are indicated by red traces and those of healthy subjects are indicated by blue traces. The baseline power spectra are presented by green traces (Figure 5**A**–**H**). An asterisk shows the significant difference between healthy subject’s vs patients (Wilcoxon signed-rank test, *p* < 0.05).

The comparison of each component power spectrum in terms of the “difference between post-stimulation and pre-stimulation” effects is shown in Figure 5. EEG power increased more in the LPL than in the LPG, RPG, and RPL regions of the brain (Figure 5A–H). The power spectra of theta, beta, and gamma bands increased significantly after noisy GVS (i.e., post-stimulation–pre-stimulation) in the LPL both during walking and standing in patients with BVH (Figure 5E,F).

## 4. Discussion

In our study, we utilized motion capture analysis and EEG recordings to delineate the post-stimulation effects of noisy GVS during standing and walking with horizontal head turning in healthy subjects and patients with BVH. By this combined approach, we demonstrated that GVS enhances the standing postural stability and improves the head rotation rhythm during walking in both subject groups. These performance improvements are possibly attributed to neuroplasticity changes induced by noisy GVS in the vestibular cortex, as indicated by signal changes in the LPG, RPG, LPL, and RPL. These areas were specifically explored in our study as they have been identified as the main vestibular cortex regions in humans together with the central sulcus and insular lobe [18,27].

The vestibular system is the sensory system that primarily contributes to the detection of angular motion and sense of spatial orientation, necessary to manage movement while maintaining balance and to stabilize postural control of the body [18,27]. Previous research has demonstrated that noisy GVS improves static balance in patients with BVH [2]. Only recently, this specific method of vestibular stimulation has shown promising results, enhancing postural and walking stability, both in older people and in patients with BVH [1,3,12,13]. Wuehr et al. proposed that the mechanism of noisy GVS effects lies in its SR nature [3]. SR is the phenomenon by which a noisy input with a mean value other than 0 (below the intensity of human perception), through chaotic numbers, may optimize the sensory nervous system and facilitate the incorporation of incoming information from the outside world [28]. The basis of this theory was established in rat and cat models [29,30], and SR has been practically applied to various sensory receptors throughout the human body, allowing more acute hearing [31] and improving the control of lower limb posture [32,33]. When SR occurs in conjunction with vestibular nerve stimulation, a very low-intensity electrical current is sufficient to assist balance function. A study by Pal et al. showed that vestibular nerve stimulation with an intensity of 0.1 mA improves the balance of patients with Parkinson’s disease [34]. Similarly, Iwasaki et al. found that 200–400 μA of noisy electrical stimulation of the vestibular nerve promotes the maintenance of the center of gravity position in healthy people and patients with bilateral vestibulopathy [2].

Conventionally, bilateral bipolar GVS enhances the firing rate of vestibular afferents by depolarization on the cathodal side and reduces their firing rate by hyperpolarization on the anodal side. Usually, the anodal stimulation site is depolarized, but the frequency and intensity of different stimulations can change the polarization to hyperpolarization [9,10,11]. Zero-mean noisy GVS has a very important advantage over conventional GVS, in that it does not induce unilateral oculomotor and postural responses. Moreover, in comparison to other suggested treatment modalities for patients with BVH, such as vestibular implants that excite the peripheral vestibular nerves through inserted probes or electrodes [34,35], noisy GVS is non-invasive and easy to apply, with fewer side effects, such as the loss of hearing related to cranial surgical operations.

Analysis of spectral changes in EEG power in the motor cortex showed that the alpha power was suppressed by GVS and that this suppression was significantly higher in healthy subjects than in patients with BVH. This may be explained by the fact that bilateral damage to the vestibular system impacts the ability to maintain body balance, particularly when patients are walking [13]. The LPG and RPG, also referred to as the primary motor area of the brain, control foot movements related to walking. Our findings of alpha band power suppression in the LPG, RPG, LPL and RPL are consistent with those of previous studies in healthy subjects during walking [15,36,37,38,39]. Spectral power analyses of theta, alpha, beta, and gamma bands showed a gradual increase in the EEG power in the RPG, LPG, LPL, and RPL, which correspond to the motor and vestibular cortices of the brain, both in healthy subjects and in patients. We speculate that these increased EEG activities may indicate neuroplasticity induced by noisy GVS in the vestibular cortex (LPL, RPL) via the peripheral vestibular system. Synaptic plasticity in the vestibular cortex involves both vestibular nuclei and cerebellar circuits. Voluntary movements such as posture, balance, and coordination are represented in cerebellar fibers parallel to Purkinje cell synapses, which trigger the vestibular nuclei [40]. In turn, the vestibular nuclei send excitatory inputs to the motor cortex (i.e., extensor motor neurons of the legs or feet) [41]. The stimulation of neurons in the vestibular cortex probably induces neuroplasticity changes that enhance postural stability [42]. By measuring COP sways, Fujimoto et al. suggested that noisy GVS enhances postural stability for 4 h after discontinuing stimulation, indicating that post-stimulation effects and vestibular neuroplasticity may exist [12,14]. Through vestibular spinal reflex, standing stability improved in our subjects. Our findings are in contrast to those of Helmchen et al. [43], who observed no brain responsiveness during imperceptible noisy GVS by functional magnetic resonance imaging. This difference may be attributed to the fact that our experiments were performed during movement, with EEG signals being recorded in real time. Moreover, noisy GVS has been shown to improve sensory neuron sensitivity and enhance vestibular sensory afferent inputs during balancing tasks [1,12,13], therefore, any related cortical changes are more likely to be observed during functional tasks rather than when lying down for prolonged image acquisition. 

Notably, the behavioral and EEG results of our study showed that, with noisy GVS, the synchronization and precision of the horizontal head rotation rhythm was more pronounced in healthy subjects than in patients. Although both patients and healthy subjects reduced the head yaw error, healthy subjects showed greater improvements. The intact peripheral vestibular afferents may explain the more pronounced enhancement in healthy subjects. Our findings are opposite to those of previous studies that suggested that individuals with elevated vestibular motion perception thresholds, i.e., patients with BVH, would benefit more from noisy GVS [44,45]. However, these previous study results were mostly based on eyes-open, quiet stance experiments. Instead, our experiment combined multiple motor tasks, including walking with a 2 Hz head yaw, and optimal stimulation location frequency may vary due to different sensory-motor demands. Furthermore, we observed that, after noisy GVS, the power of beta and gamma bands increases in the somatosensory cortex (LPG and RPG) of healthy subjects during walking. These findings are consistent with those of several previous studies on GVS [15,16,18]. These earlier studies have demonstrated the post-stimulation effects of GVS in healthy subjects who use facial stimuli, albeit in sitting positions [18,46]. In contrast, our study utilized a more realistic experimental scenario, including both walking and standing conditions. In addition, we showed that the EEG power of theta, alpha, beta, and gamma bands increases significantly after noisy GVS in the vestibular cortex (LPL) both in healthy subjects and in patients, in line with the results of previous studies on GVS and gait-training [5,15,16,47,48]. The decrease, damage or decreased functionality of the vestibular system bilaterally affects the brain, making it difficult in maintaining body balance, particularly in patients during walking. This explains the observation of less mu rhythm suppression in patients than in healthy controls. Several studies have shown that the post-stimulation effects of GVS and tDCS increase the EEG power of theta, alpha, and beta bands in the frontal, temporal, posterior, parietal, and occipital lobes in normal subjects [18,46,49,50,51,52], in agreement with our findings in healthy subjects. By using ICA with dipole source localization for EEG analysis, our method goes beyond those used in previous studies, resulting in cleaner EEG signals; thus, a more precise spatial temporal resolution in the vestibular cortex could be determined.

This study had some limitations. First, we recruited a limited number of subjects, and we did not perform vestibular myogenic evoked potentials (VEMP) to assess the otolithic system. Because the aim of this study was to investigate the EEG signal changes after noisy galvanic stimulation during 2 Hz horizontal head movements, we only performed a caloric test for the evaluation of bilateral horizontal semicircular canals. Second, we did not use a control group with sham stimulation, and pre-stimulation trials might carry over some habituation effects to the post-stimulation trials. Because the stimulation intensities used in the pre-stimulation trials were sensory-imperceptible, we believe the habituation effects were minimal. In the future, we will conduct experiments with sham controls to further investigate the habituation effects of noisy GVS. Third, the effects of noisy GVS on posture stability were investigated under a laboratory setting, with controlled conditions, and a regular 2 Hz stimulus, as opposed to a real setting with more complex stimuli. Future real-world studies are required to confirm our findings. Designing new paradigms for imaging the human brain during walking, such as mobile brain-body imaging, will provide further insight regarding GVS effects.

## 5. Conclusions

EEG recordings with dipole source localization provide the unique ability to evaluate neural activities in patients with BVH. Noisy GVS changed brain activities both in healthy individuals and in patients with BVH, both when walking with 2 Hz horizontal head rotation and while standing. Our behavioral and EEG results reveal that noisy GVS improves postural stability in healthy subjects, as well as in patients, and this improvement could be due to the induction of neuroplasticity in the vestibular cortex. Noisy GVS may be used as a non-invasive adjuvant therapy for patients with BVH.

## Figures and Tables

**Table 1:** Demographic data for patients with bilateral vestibular hypofunction (BVH).

	Sex	Diagnosis	Onset Time	Clinical Presentations	Training
P1	F	idiopathic BVH	2.5 m	Unsteady gait, dizziness	NA
P2	F	idiopathic BVH	1.5 m	Unsteady gait, dizziness	NA
P3	F	idiopathic BVH	2 m	oscillopsia	1 m
P4	F	idiopathic BVH	2 m	Hearing loss, vertigo, unsteady gait	1 m
P5	F	idiopathic BVH	1 m	Unsteady gait, dizziness	1 m
P6	F	idiopathic BVH	2.5 m	Dizziness, hearing loss, unsteady gait	0.5 m
P7	F	idiopathic BVH	1.5 m	Vertigo, unsteady gait	NA

## Abbreviations

GVS	galvanic vestibular stimulation
EEG	electroencephalography
SR	stochastic resonance
RMS	root mean square
COP	center of pressure
ICA	independent component analysis
BBS	blind source separation
BVH	bilateral vestibular hypofunction
ERSP	event-related spectral perturbation
LFG	left frontal gyrus
RFG	right frontal gyrus
LPG	left precentral gyrus
RPG	right precentral gyrus
LPL	left parietal lobe
RP	right parietal lobe
OL	occipital lobe

## References

[1] Wuehr M., Decker J., Schniepp R. (2017). Noisy galvanic vestibular stimulation: An emerging treatment option for bilateral vestibulopathy. J. Neurol..

[2] Iwasaki S., Yamamoto Y., Togo F., Kinoshita M., Yoshifuji Y., Fujimoto C., Yamasoba T. (2014). Noisy vestibular stimulation improves body balance in bilateral vestibulopathy. Neurology.

[3] Wuehr M., Nusser E., Decker J., Krafczyk S., Straube A., Brandt T., Jahn K., Schniepp R. (2016). Noisy vestibular stimulation improves dynamic walking stability in bilateral vestibulopathy. Neurology.

[4] Gittis A.H., du Lac S. (2006). Intrinsic and synaptic plasticity in the vestibular system. Curr. Opin. Neurobiol..

[5] Lopez C., Blanke O., Mast F. (2012). The human vestibular cortex revealed by coordinate-based activation likelihood estimation meta-analysis. Neuroscience.

[6] Zu Eulenburg P., Caspers S., Roski C., Eickhoff S.B. (2012). Meta-analytical definition and functional connectivity of the human vestibular cortex. Neuroimage.

[7] Goldberg J., Smith C.E., Fernandez C. (1984). Relation between discharge regularity and responses to externally applied galvanic currents in vestibular nerve afferents of the squirrel monkey. J. Neurophysiol..

[8] Minor L.B., Goldberg J.M. (1991). Vestibular-nerve inputs to the vestibulo-ocular reflex: A functional-ablation study in the squirrel monkey. J. Neurosci..

[9] Yamamoto Y., Struzik Z.R., Soma R., Ohashi K., Kwak S. (2005). Noisy vestibular stimulation improves autonomic and motor responsiveness in central neurodegenerative disorders. Ann. Neurol..

[10] Pan W., Soma R., Kwak S., Yamamoto Y. (2008). Improvement of motor functions by noisy vestibular stimulation in central neurodegenerative disorders. J. Neurol..

[11] Soma R., Kwak S., Yamamoto Y. (2003). Functional stochastic resonance in human baroreflex induced by 1/f-type noisy galvanic vestibular stimulation. In: Fluctuations and Noise in Biological, Biophysical, and Biomedical Systems. Int. Soc. Opt. Photonics.

[12] Fujimoto C., Yamamoto Y., Kamogashira T., Kinoshita M., Egami N., Uemura Y., Togo F., Yamasoba T., Iwasaki S. (2016). Noisy galvanic vestibular stimulation induces a sustained improvement in body balance in elderly adults. Sci. Rep..

[13] Fujimoto C., Egami N., Kawahara T., Uemura Y., Yamamoto Y., Yamasoba T., Iwasaki S. (2018). Noisy galvanic vestibular stimulation sustainably improves posture in bilateral vestibulopathy. Front. Neurol..

[14] Iwasaki S., Karino S., Kamogashira T., Togo F., Fujimoto C., Yamamoto Y., Yamasoba T. (2017). Effect of noisy galvanic vestibular stimulation on ocular vestibular-evoked myogenic potentials to bone-conducted vibration. Front. Neurol..

[15] Seeber M., Scherer R., Wagner J., Solis-Escalante T., Müller-Putz G.R. (2014). EEG beta suppression and low gamma modulation are different elements of human upright walking. Front. Hum. Neurosci..

[16] Bruijn S.M., Van Dieën J.H., Daffertshofer A. (2015). Beta activity in the premotor cortex is increased during stabilized as compared to normal walking. Front. Hum. Neurosci..

[17] Lee M.H., Durnford S.J., Crowley J.S., Rupert A.H. (1997). Visual vestibular interaction in the dynamic visual acuity test during voluntary head rotation. Aviat. Space Environ. Med..

[18] Kim D.J., Yogendrakumar V., Joyce Chiang E.T., Wang Z.J., McKeown M.J. (2013). Noisy galvanic vestibular stimulation modulates the amplitude of EEG synchrony patterns. PLoS ONE.

[19] Delorme A., Makeig S. (2004). EEGLAB: An open source toolbox for analysis of single-trial EEG dynamics including independent component analysis. J. Neurosci. Methods.

[20] Makeig S., Debener S., Onton J., Delorme A. (2004). Mining event-related brain dynamics. Trends Cogn. Sci..

[21] Jung T.P., Makeig S., Westerfield M., Townsend J., Courchesne E., Sejnowski T.J. (2000). Removal of eye activity artifacts from visual event-related potentials in normal and clinical subjects. Clin. Neurophysiol..

[22] Makeig S., Inlow M. (1993). Lapses in alertness: Coherence of fluctuations in performance and EEG spectrum. Electroencephalogr. Clin. Neurophysiol..

[23] Efron B., Tibshirani R.J. (1994). An Introduction to the Bootstrap.

[24] Benjamini Y., Hochberg Y. (1995). Controlling the false discovery rate: A practical and powerful approach to multiple testing. J. R. Stat. Soc. Ser. B.

[25] Ko L.W., Shih Y.C., Chikara R.K., Chuang Y.T., Chang E.C. (2016). Neural mechanisms of inhibitory response in a battlefield scenario: A simultaneous fMRI-EEG study. Front. Hum. Neurosci..

[26] Chikara R.K., Chang E.C., Lu Y.C., Lin D.S., Lin C.T., Ko L.W. (2018). Monetary reward and punishment to response inhibition modulate activation and synchronization within the inhibitory brain network. Front. Hum. Neurosci..

[27] Guldin W., Grüsser O. (1998). Is there a vestibular cortex?. Trends Neurosci..

[28] Collins J., Chow C.C., Imhoff T.T. (1995). Stochastic resonance without tuning. Nature.

[29] Collins J.J., Imhoff T.T., Grigg P. (1996). Noise-enhanced information transmission in rat SA1 cutaneous mechanoreceptors via aperiodic stochastic resonance. J. Neurophysiol..

[30] Fallon J.B., Carr R.W., Morgan D.L. (2004). Stochastic resonance in muscle receptors. J. Neurophysiol..

[31] Zeng F.G., Fu Q.J., Morse R. (2000). Human hearing enhanced by noise. Brain Res..

[32] Priplata A.A., Niemi J.B., Harry J.D., Lipsitz L.A., Collins J.J. (2003). Vibrating insoles and balance control in elderly people. Lancet.

[33] Reeves N.P., Cholewicki J., Lee A.S., Mysliwiec L.W. (2009). The effects of stochastic resonance stimulation on spine proprioception and postural control in chronic low back pain patients. Spine.

[34] Pal S., Rosengren S.M., Colebatch J.G. (2009). Stochastic galvanic vestibular stimulation produces a small reduction in sway in Parkinson’s disease. J. Vestib. Res..

[35] Guyot J.P., Gay A., Izabel K.M., Pelizzone M. (2012). Ethical, anatomical and physiological issues in developing vestibular implants for human use. J. Vestib. Res..

[36] Jasper H., Penfield W. (1949). Electrocorticograms in man: Effect of voluntary movement upon the electrical activity of the precentral gyrus. Arch. Psychiatr. Nervenkrankh..

[37] Crone N.E., Miglioretti D.L., Gordon B., Sieracki J.M., Wilson M.T., Uematsu S., Lesser R.P. (1998). Functional mapping of human sensorimotor cortex with electrocorticographic spectral analysis. I. Alpha and beta event-related desynchronization. Brain.

[38] Miller K.J., Leuthardt E.C., Schalk G., Rao R.P., Anderson N.R., Moran D.W., Miller J.W., Ojemann J.G. (2007). Spectral changes in cortical surface potentials during motor movement. J. Neurosci..

[39] Severens M., Nienhuis B., Desain P., Duysens J. Feasibility of measuring event related desynchronization with electroencephalography during walking. Proceedings of the 2012 Annual International Conference of the IEEE Engineering in Medicine and Biology Society.

[40] De Zeeuw C.I., Hansel C., Bian F., Koekkoek S.K., Van Alphen A.M., Linden D.J., Oberdick J. (1998). Expression of a protein kinase C inhibitor in Purkinje cells blocks cerebellar LTD and adaptation of the vestibulo-ocular reflex. Neuron.

[41] Grillner S., Hongo T. (1972). Vestibulospinal effects on motoneurones and interneurones in the lumbosacral cord. Progress in Brain Research.

[42] Grassi S., Pettorossi V.E. (2001). Synaptic plasticity in the medial vestibular nuclei: Role of glutamate receptors and retrograde messengers in rat brainstem slices. Prog. Neurobiol..

[43] Helmchen C., Rother M., Spliethoff P., Sprenger A. (2019). Increased brain responsivity to galvanic vestibular stimulation in bilateral vestibular failure. Neuroimage Clin..

[44] Galvan G.R., Clark T., Mulavara A., Oman C. (2018). Exhibition of stochastic resonance in vestibular tilt motion perception. Brain Stimul..

[45] Priesol A.J., Valko Y., Merfeld D.M., Lewis R.F. (2014). Motion perception in patients with idiopathic bilateral vestibular hypofunction. Otolaryngol Head Neck Surg..

[46] Wilkinson D., Ferguson H.J., Worley A. (2012). Galvanic vestibular stimulation modulates the electrophysiological response during face processing. Vis. Neurosci..

[47] Ertl M., Moser M., Boegle R., Conrad J., zu Eulenburg P., Dieterich M. (2017). The cortical spatiotemporal correlate of otolith stimulation: Vestibular evoked potentials by body translations. NeuroImage.

[48] Lobel E., Kleine J.F., LEROY-WILLIG A., VAN DE MOORTELE P.F., Bihan D.L., GRÜSSER O.J., Berthoz A. (1999). Cortical areas activated by bilateral galvanic vestibular stimulation. Ann. N. Y. Acad. Sci..

[49] Song M., Shin Y., Yun K. (2014). Beta-frequency EEG activity increased during transcranial direct current stimulation. Neuroreport.

[50] Ray W.J., Cole H.W. (1985). EEG alpha activity reflects attentional demands, and beta activity reflects emotional and cognitive processes. Science.

[51] Keeser D., Padberg F., Reisinger E., Pogarell O., Kirsch V., Palm U., Karch S., Möller H.J., Nitsche M., Mulert C. (2011). Prefrontal direct current stimulation modulates resting EEG and event-related potentials in healthy subjects: A standardized low resolution tomography (sLORETA) study. Neuroimage.

[52] Stam C., Montez T., Jones B., Rombouts S., Van Der Made Y., Pijnenburg Y., Scheltens P. (2005). Disturbed fluctuations of resting state EEG synchronization in Alzheimer’s disease. Clin. Neurophysiol..

